# On the relevance of (the New) Phenomenology to an ethics of health promotions: toward a prudent balance of understanding and explanation

**DOI:** 10.1186/s13010-023-00135-7

**Published:** 2023-07-12

**Authors:** Christina Röhrich, Nikola B. Kohls, Eckard Krüger, James Giordano

**Affiliations:** 1 Program in Integrative Health Promotions, Coburg University of Applied Sciences, Coburg, Germany; 2 Department of Geriatric Medicine, Hospitals Hochfranken, Naila, Germany; 3 Department of Neurology; and Pellegrino Center for Clinical Bioethics, Georgetown University Medical Center, Washington, DC, USA

**Keywords:** Health promotion, Ethics, New Phenomenology, Subjectivity, Objectivity

## Abstract

The field of health promotions faces considerable ethical and programmatic challenge – and we believe opportunity – in addressing the relative normativity of the concept(s) of health and its professional handling. To date, distinctions of objective and subjective indicants of “health” have fostered normative tension(s) within the utilitarian ethics of health promotions, which we opine to be anathema to the ultimate goal(s) of attaining and sustaining healthy individuals and societies. Objective and subjective metrics and values should be reconciled, as reciprocal and complementary on both idiosyncratic and systemic levels. In this light, we propose that a philosophical and ethical approach, based primarily upon Schmitz’s New Phenomenology and its specific understanding of subjectivity could afford epistemological bases for non-normative engagement of health promotion within a structural–functional framework of ethics. We dialectically address its potential benefit, limitations and value for health promotion and health care ethics and present an approach which points towards a more substantial encouragement of diversity through the legitimation of subjectivity.

## Introduction

### Normativity in health promotion

Health promotion is becoming increasingly recognized as a discipline and set of practices that are important to preventive, empowering, and resources-oriented sustainable approaches in social contexts of biomedicine. However, translating health promotion theory and concepts into constructive applied practice poses opportunities, as well as challenges, and risks, particularly when considering and engaging socio-cultural, economic and ethical variation on local and global scales. In part, this is because health goals and endpoints are most often established and justified epidemiologically in functionalized cost-effort interventionist frameworks, which generally reflect and favor collective utility; whereas individual dimensions of health such as well-being, thriving, and/or flourishing tend to recede from primary focus.

This prompts questions of utilitarian approaches to population health, viz.—what is/are of greatest health promotional benefit for the greatest number of individuals? Queries of this sort often establish systematization of current and developing social norms that presume, yet supersede—if not overlook—the needs, demands and sometimes even values of individuals. Although ideals of self-determination, self-efficacy and autonomy are central social values within health promotions’ discourses, personal responsibility while advocated, is implicitly supported – for example by means of “nudging” as a socio-technological technique – with aims of producing desired behaviors that are considered to be advantageous to/for health economics. Such desired behaviors are in turn related to epidemiologically based variables, thus contextualizing personal responsibility within consensus-based constructs of empirically-based "right”, as well as righteous behavior. This creates a paradox, and perhaps a dilemma in that the establishment of biasing norms – while fostering ingroup coherence – may instigate stigmatization and social exclusion, which certainly must be viewed as diametrically opposed to the intention of promoting (bio-psychosocial) health in an inclusive manner for all individuals as meaningful constituents of the collective groups in which persons are embedded.

## Discussion

### Objectivity, normativity, and the value of subjectivity

Since current theories and activities of health promotion are based upon scientific method(s) and views –notably in accordance with the prevailing biomedical paradigm – interest is focused on objectively measurable parameters, and subjective domains tend to be regarded with lesser import. Although qualitative research approaches explicitly address subjective dimensions, the results of such studies are also utilized to inform generalizable theories and practices [1]. Hence, the focus is primarily on objective parameters for influencing health determinants (for research) that are translatable to monitor and affect target parameters of various interventions.

Yet, the philosophical-anthropological foundation of health promotion, while indubitably important to epistemic, socio-culturally, and ethically relevant understanding of existential aspects of the human condition and predicament (of injury, disease, sickness) is rarely addressed, and even less frequently engaged in research and practice. This is not due to a paucity of discourse; to the contrary, discussions of health and its promotion have been richly engaged in dialogues of history and humanistic ideas [2]. Such dialogue has been dialectical: means of promoting health of individuals and groups have been posited and viewed as reasonable, important, and honorable intentions, and laudable efforts. However, it is important to note that the criteria and definition of what constitutes “health”, its implicit norms, and the desirability of such characteristics (and therefore what is unhealthy, abnormal, and implicitly if not explicitly undesirable) can yield considerable power, and can be employed to evoke biopolitical manifestations [3, 4]. Such precepts can become associated with, and influential to civic duties, and may blur boundaries between public and private goals, responsibilities, and obligations [2, 4].

In this context, it is important to critically reflect on the relative value of normativity, and to establish an understanding of health promotion as entailing and obtaining an educational and empowerment process, and in these ways can – and should—contribute to the health and wellness of both individuals and their collective(s). Undergirding any approach to health promotion is an understanding and definition of health. Herein, we believe that it is useful and helpful to distinguish between naturalistic and normativistic conceptions of health. The naturalistic view considers health and disease to be value-free; while the normativist perspective regards these as value-associated categorizations that are ultimately related to functional endpoints, without which assessment of health (and unhealth) cannot be made [5]. The analytic-naturalistic view refers to biological characteristics that reflect objective aspects of health and disease, and which "…relates bodies, organs, and human behavior to an empirically naturalistically determined supra-individual norm" ([5], p. 42). This objective aspect of health is prevalent in health promotion, and is characteristically used as a basis for evidence-based intervention(s).

However, it is also important – and we believe necessary – to appreciate and engage a complementary understanding (albeit a somewhat contentious one), of a "subjectivist-holistic concept[ion] of health, shaped by phenomenological-existential thinking, which equates the achievement of personal goals with the attainment of happiness" ([5]), p. 42; see also [6], and [7]). From this idiosyncratic and subjectivist position, which links health to well-being and perceived quality of life, a relational position, which focuses on competence and goal achievement, can be identified. One criterion of the subjective aspect in normativism obtains that not every deviation from a given normative measure must be classified as "un-well", per se.

The World Health Organization (WHO) concept of health can—and we opine, should—be viewed through this subjectivist-oriented lens. For example, Schröder-Bäck [5] draws attention to the fact that approaches that emphasize subjective, idealistic characteristics must be regarded critically, given that boundaries are often blurred, and demarcation of interventions as health promotional, therapeutic, or enhancing becomes difficult in light of relative subjectivity. Thus, the idealistic concept of health (e.g.- as espoused by WHO,) would remain vague, and perhaps unattainable, both in conceptual and practical implementation.

We find this to be errant at least, and wasteful and potentially harmful at worst. The call, and identified need for the integration of naturalistic and normativistic approaches is understandable; but while it seems that subjective dimensions cannot be fully captured, we argue that this does not infer that they be disregarded [8]. This harkens consideration of the distinctions between (first person) understanding (i.e.- *verstehen*) and objective explanation (i.e.- *erklären*), as introduced by Droysen [9] and expounded upon by Dilthey [10]. Indeed, idiosyncratic factors are often elusive, and even mixed methods mandate the use of some instrument(s) to attempt to “objectify the subjective” dimensions of evidence that can be used to evaluate and affect existential domains of the human condition and predicament [11].

Such (a set of) instruments would first need to be presented linguistically, because individual experience must be qualitatively represented (primarily) by appropriate terminology and relevant explanatory viability, validity, and value. As Dilthey [10] noted, *verstehen* (understanding) is seemingly antithetic, and often unapprehendable from a purely scientistic (if not positivistic) perspective. The scientific paradigm, while certainly requiring and advocating explanatory value, in the main focuses and relies upon quantitative parametrization, predictability, and replicability of natural events and processes. Thus, health – of the individual and as a generalizable construct—becomes an object of cognition: both idiosyncratically (viz- how the individual feels), and systemically (i.e.- how various states and conditions are regarded, and "the human being becomes an object of the natural scientific gaze to be standardized" ([12], p. 293). A set of instruments for apprehending pure subjectivity – for example to access and assess the subjectivity of spiritual experiences, values and beliefs in a non-reductionist way – has yet has not been developed within the regnant objectivist model for methods of scientific investigation and examination. This prompts a turn to epistemological concepts and philosophical-anthropological foundations.

### Value desiderata of the bio-psychosocial model

The biopsychosocial approach advocated by Engel [13] deconstructs some of the monocausal rigidity of the longstanding biomedical model in its representation of, and attendance to reciprocal and multidimensional factors of physical embodiment and (physical-socio-ecological) embeddedness [14, 15]. Still, the biopsychosocial model retains certain reductionist elements in that it tends to conjoin (and/or conflate) individual domains like putative modules, thus providing only a patchwork anthropology that is frequently misunderstood, and regularly misused as rationale for interventional over-provision and therapeutic arbitrariness (which are axiomatically antithetic to current calls for personalized precision care (vide infra). In this light, it remains unclear how the interfaces and/or intersections of the biological, psychological, and social domains might shape, and be shaped by human experiences, values and beliefs. We see this not merely as a limitation or challenge, but rather as an opportunity for continued discourse to employ and expand the bio-psychosocial construct to effect a more complete and authentic understanding of the human condition that incorporates, but further evolves and broadens the extant biomedical model by introducing a dimension capable of addressing individual subjectivity as constituent to collective constructs of health, and objective metrics ad means of health promotion.

Toward such ends, we posit that a complementary approach is necessary, which engages a form of hermeneutics to apprehend and appreciate the subjective, phenomenologic aspects of experience (see [16]), and an adaptive reflective equilibrium [17] to develop and articulate objective methods for promoting, restoring and sustaining individual and collective health. To be sure, putting the conceptual dimensions of this model into real-world practice represents a work-in-progress. Critical to such enterprise is the need to assess the ethical domains, realities, and exigencies that health – and thus health promotion—entails and requires.

Any authentic conceptualization of health necessitates insight, sensitivity, and responsiveness to both subjective experience, needs, and values, and the objective dimensions that are useful for establishing practical norms and standards for individuals in particular collectives. We opine that moving from the subjective to the objective, and appreciating the reciprocity of these domains is important because to objectify health – and thereby establish bases for its promotion – requires some codification of phenomenological experience within definable—but nonetheless surrogate – parameters. Such surrogation occurs in the objectification of subjective experience, and in the instantiation of collective norms and standards that are construed from individual necessities and values. However, this implies an exploration of the relation between subjectivity and objectivity at some depth. While science, in particular the domain of natural science, has shaped our view of life and to some degree even infiltrated our perception of being in the world, it would seem almost intuitive to regard subjectivity as the remnants of what cannot be objectified. At best, subjectivity and objectivity would be regarded as complementary, maybe even mutually exclusive like the reverse sides of a coin. This poises the question how these two domains interact in real life and also if there is a general bias towards objectivity as being more legitimate and holding the primacy over subjectivity. Schmitz [18] regards this as the abstraction base, which acts like a filter between perception and conviction. According to Schmitz, “The (…) experience of life (…) is not a landing place that can be headed straight for but is only accessible through the filter of (…) perspectives [on a basis of abstraction]" [18, p. 13]. This of course, holds extensive implication regarding our understanding of health and illness alike.

The philosopher Hans-Georg Gadamer [19] has likened being healthy to "self-obliviousness" suggesting that the experience of a healthy self resides beneath the threshold of self-perception so that the individual can live his/her life in an embedded way, which seems to have some resemblance to a sense of flow. In contrast, being unwell presents itself through a sense of objectification in that the painful/unwell part stands out of the state of self-obliviousness, even to the extent that the individual will designate it with a possessive pronoun (e.g. “*my* tooth hurts”). In a peculiar way the unwell part seems to depart from a sense of self into a state of reification. As a consequence, Gadamer suggests that a state of wellbeing or un-wellbeing can only be understood as relational, while, essentially, the individual needs to be put into relation to herself/himself. In some medical systems this is a practice with longstanding tradition, e.g., in Traditional Chinese Medicine—employing a system of analogies and relations tachycardia is not a number of heart beats per minute but per breath cycle of the individual.

According to Gadamer, "health conceals itself (…) it comes to light in a feeling of well-being, and even more in the fact that we are (…) self-oblivious for the sake of well-being, and hardly feel even strains and efforts-that is health” ([19], p. 143). This does not infer that health is obtuse and cannot be qualified and even quantified to some extent in objective ways and terms. Rather, it reinforces that these objectifications (i.e.- explanations) must identify, account for, and accord subjective dimensions as known (i.e.- understandings) and explicated by individuals – both singularly and in collective.

Thus, if a health care and/or health promotions’ approach or system seeks to develop ethics that advocate and sustain authentic goods (as relevant to those individuals that are the subjects of these approaches moral regard), any calculus of utility must engage a balanced consideration of both objective and subjective domains. Utilitarian approaches to health promotion are common (see, for example, [20–24]). But collectivist utilitarian approaches dominate population-based public health ethics, and thus individual notions of health (and its subjective experience and definition) are characteristically superseded by aggregate (and usually quantitative) conceptualizations. This even applies for much appraised target group orientation. Yet, as previously noted, this seems contrary (if not wholly anathema) to emphatic calls for, and current trends toward personalized and precision healthcare.

How then might these be reconciled? The contemporary philosopher Hermann Schmitz has described a philosophical orientation – the New Phenomenology – which preserves the notion and value of subjectivity. In Schmitz's phenomenology, explanations of subjective experience enable depiction of objectifiable precepts of individuals’ (and collectives of individuals’) perception(s) of the felt body and being-in-the-world. Central to the New Phenomenology is validation and legitimization of subjective experience as being inaccessible to objectifying sciences absent the translation of understanding (*verstehen*) to explanation (*erklären*).

This philosophical approach strives to make the "basic experiences of human existence" in terms of the “involuntary aspects of life” ([25], p. 7) for human beings accessible and expressible. For Böhme [26], life experiences (and their non-measurable phenomena of corporeality, feelings, and bounded subjectivity) are closed to purely objective scientific inquiry and quantification. Böhme has described Schmitz's approach as a "fundamental critique of our scientific-technical way of life" [26], but not in a derogatory or denigrative way. Rather, the basis of critique lies in the element of criticality; simply that the subjective domain is critical (i.e.- essential) to any authentic and realistic attempt(s) and approach(es) to objectifying the experiential aspects of health and well-being [27].

### Subjectivity in the New Phenomenology

The conventional understanding of subjectivity is that it entails and obtains the personal experience of an individual. This explicates its containment – and relative constraint. Because it is true that subjective experience is transparent only in the first-person-perspective, it demands a phenomenological approach to access these experiences and facilitate interpersonal communication about subjective experiences. Nevertheless, recognition of the constrained properties of individual experience has fostered implication that subjectivity is “inferior” to objectivity [28]. Schmitz in contrast, proposes a much more radical understanding of subjectivity. Following Schmitz, a single person is only able to perceive a subjective experience. In order for this to become objective, a gradual process of emancipation like ‘peeling off layers of subjectivity’ is required, which by necessity is facilitated and moderated by language. According to Schmitz, subjective experience originates in a state of affective corporeal experience (viz., in German: *affektive leibliche Betroffenheit*). From this state of preverbal subjective human experience, the person will gradually begin to reflect upon the phenomenal aspects, eventually elaborating upon them until a state of complete emancipation from this subjective experience is reached. The experience may now be phrased in rather general terms that are relatable to anyone who has had similar experiences. So, the subjective quality of the experience becomes diluted into something much more general (i.e., objective) and much less personal (i.e.- subjective). Here, Schmitz concludes, that subjectivity -as the cradle of objectivity – retains primacy, while objectivity becomes a secondary occurrence. The common sense of Western intellectual culture would state otherwise: namely, that primacy is nested in objective facts. However, Schmitz' terminology instead posits the value of both subjective facts and objective facts.

According to Schmitz [29], a subjective fact is one that only a single person can state because only that one person experiences it in an embodied way. Here, Schmitz distinctly differentiates between the body as a three-dimensional physical object (i.e.- *Körper*), which itself is unable to experience or feel anything, and the being inhabiting the lived body (i.e.- *Leib)*, who is phenomenologically engaged in the subjectivity of the experience. To emphasize this difference within the terminology of his approach to phenomenology, Schmitz uses the German term ‘*Leib*’ to refer to the lived aspect of the body. Schmitz et al. [30] describe this aspect:

*First of all, it is important not to reify the felt body and dualistically oppose it to the ‘material body’ [Körper in German], although Schmitz’ use of the noun “Leib” might sometimes create this misleading impression. Much rather, the felt body is a feeling body—its mode of existence cannot be separated from its becoming manifest to the conscious subject in specific kinds of corporeal feeling. These corporeal feelings are crucially distinct from what usually gets described under the term ‘bodily sensations’ (in psychology or the analytical philosophy of mind): the feeling body becomes manifest in holistic corporeal stirrings such as vigour and languidness, in one’s being corporeally gripped by emotions and room-filling atmospheres, and equally in one’s corporeal orientation in the world in contexts of perception, action and spatial navigation. Moreover, the feeling body presents an absolute location of subjective orientation and opens the dimension of a predimensional, surfaceless space.*

Schmitz provides several examples, e. g. fright, pain, grief, joy, etc.; any of which will manifest primarily as an affective state of the felt body (*Leib*). Thus, while *Leib* and *Körper* intersect, they do not necessarily have the same borders and limitations. This can be seen in patients following an amputation, whereby a bodily part may not be present, yet the sensation of the missing limb may still persist (i.e.- phantom sensations); thus, the lived experience of the body (Leib) remains, although the physical aspect of (a part of) the body (*Körper*) does not. In contrast, patients with neuropathic pathology of the extremities (e.g. diabetogenic neuropathy) very often lack sensation in body parts they have. Here, the body (*Körper*) is present, while the lived experience of its presence (viz.- *Leib)* is not. Schmitz claims that the primary experience of *Leib* serves as the forum for any consciousness of embodiment. Here, it is important to note that subjective fact is the basis of (1) phenomenal experience, (2) reflection and understanding; and (3) personal narrative. This reflective process yoked to language. Schmitz terms this process ‘personal emancipation’, and the situation of affective bodily experience he calls ‘personal regression into a state of pre-personal subjectivity’ [31]. Personal development and growth are viewed as ongoing oscillation(s) between personal regression and personal emancipation. Further, this oscillation gradually transforms subjective facts into objective facts, as the qualities of subjectivity are iteratively diffused into more objectively verbalized expression that are generalizable to others.

Subjective facts are not transparent to, or directly knowable by others but can only be communicated by language, and/or inferred by means of decoding proxy parameters such as facial expressions, or modulation and pitch of voice. To re-iterate, embodied persons are embedded in (temporal, spatial, and circumstantial) environments and ecologies, and this establishes (if not necessitates) interaction with others.

This understanding of subjectivity as well as its relation to objectivity has a number of implications, which are beyond the scope of this manuscript. However, perhaps the most important implication is for an understanding and role of normativity and diversity. The traditional understanding and primacy of objectivity must lead to a more normative understanding of health, disease, and illness, inclusive of the ethical dimensions that are inherent to, and arise from these constructs. The radical turn towards subjectivity, as suggested by Schmitz, may encourage diversity and thereby encourage respect, and welcome diversity because it legitimizes individual experience. In this way, health promotion as a discipline, as well as an ethically informed health care practice, may focus upon primary subjective experiences and subjective narratives within contexts of objective facts (of human ecology) as factors that contribute to and establish “health”.

The adage “nothing about us without us” bespeaks the necessity of subjective experience and understanding (of health, well-being, illness, etc.) to objective explanations and articulations of health promotional enterprises. Focus upon the bodily nature of the human being is a suitable antidote to simple objectification of the body. As Böhme notes: "the subject correlatively becomes a narrative construct or, as Marx said, a node of social relations. It loses the body as the source of the self" ([26], p. 239). Moreover, the New Phenomenology implies a social and action-oriented concept of health, based on felt bodily experience (*leiblicher Erfahrung)*. This can be well aligned both with concepts of salutogenesis, and resilience theories, as the focus is on the constant emergence of health through a maturational process of personal regression and emancipation.

This suggests that, strictly speaking, there can be no targeted, group-specific health promotion without address of—and respect as well as appreciation for—the subjectivity of each person in the group. Thus, there can be no inclusive form of health promotion as based on identity politics that could justly balance the needs of all groups, while still taking due account of individual differences within the group. In this respect, neglecting subjective needs, desires and values in favor of purely normative approaches to health promotion inevitably leads to paternalism and exclusion. For this reason, we consider it non- permissible to apply a purely public health ethic to the field of health promotion. Rather, we propose a more balanced and layered view, primarily focusing on the individual as the primary domain of importance, while concomitantly attending to collectivist group-community obligations, needs and demands, and as based on indispensable formal rights, duties and rules. Health promotion that programmatically considers subjectivity in such a way would develop an anthropology, allowing both target variables to change and methodological approaches to evolve.

## Conclusion

### Phenomenologic elements of a structural–functional ethics of health promotion

Philosopher and cognitive scientist Owen Flanagan [32] has described ethics as “human ecology”, in its literal sense (i.e.- from the Greek, *oikos*—household; *logos* – logical reasoning) as a rational approach and accounting of the environments and resources essential to human relationships, survival and flourishing. Apropos this definition, an authentic ethics of health promotion should identify, address, and seek to improve the realities of humans-in-ecology, and in this way appreciate the contingencies and exigencies of the individual, *as well as* the collective.

**Fig. 1:**
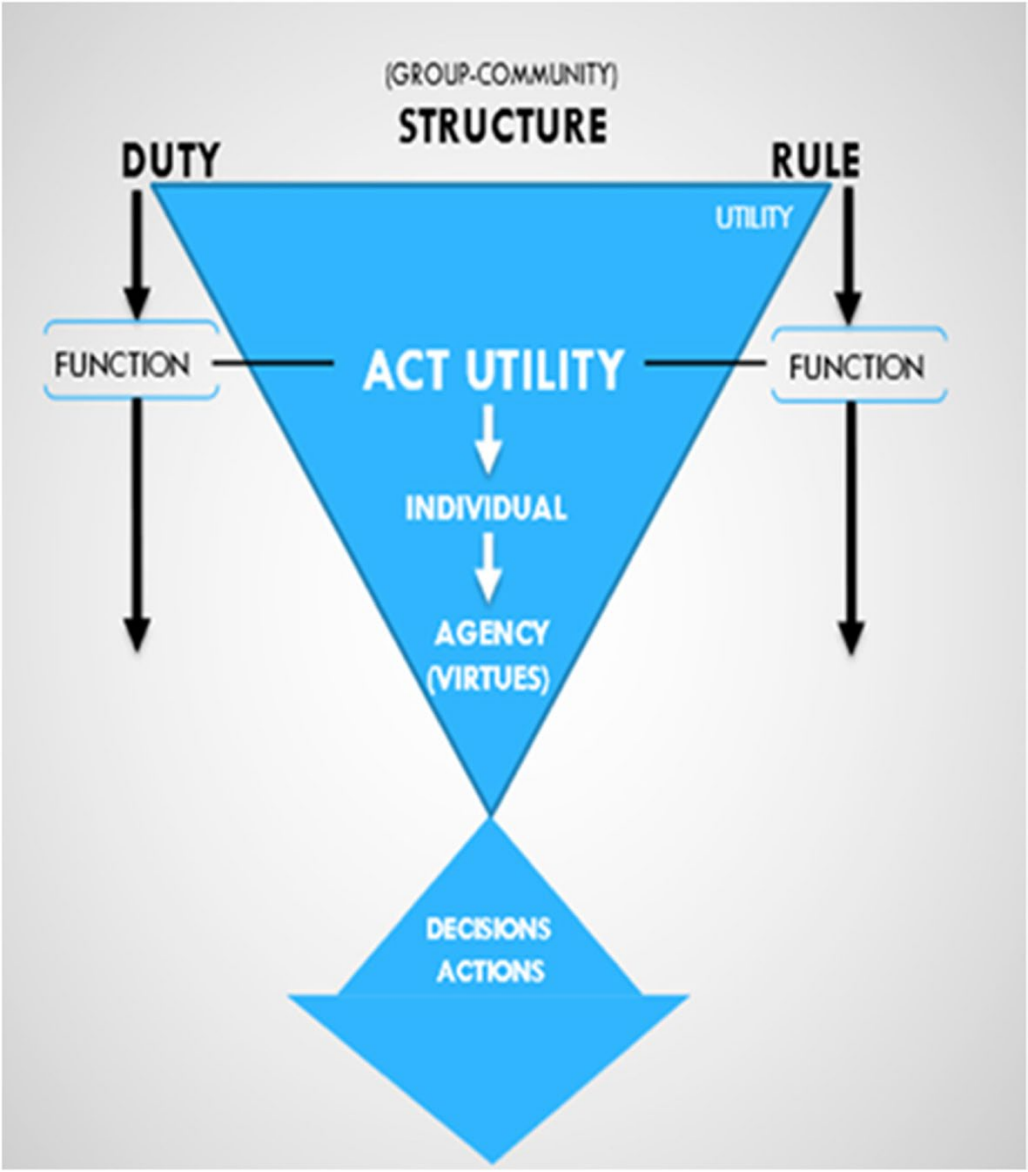
Schematic depiction of a structural–functional framework of ethics in which deontological and rule utilitarian aspects constitute the professional structural elements, and functional articulation is engaged through act utility and agentic decisions and action(s) within the context(s) of a particular collective (Adapted from: Applewhite, Giordano, Girton, Procaccino [35], with permission)

We have previously described a structural–functional ethics whereby duties and rules of establish the primary ethical structure ([33–35]; see Fig. 1). However, understanding the basis of the individual enaction is critical. And so, the functional component of ethics begins with an individual's capability to understand and enact relative goods within the scope of duties and rules. That scope of duties and rules also upholds individual and community care obligations to meet the needs and values of the persons and group respectively involved and affected. Ultimately, function is executed by agents’ actions-in-practice that obtain alignment of individuals’ moral compass with the duties, outcomes, and service—both to other individuals and the group at-large—as the subjects of moral responsibility.

**Fig. 2:**
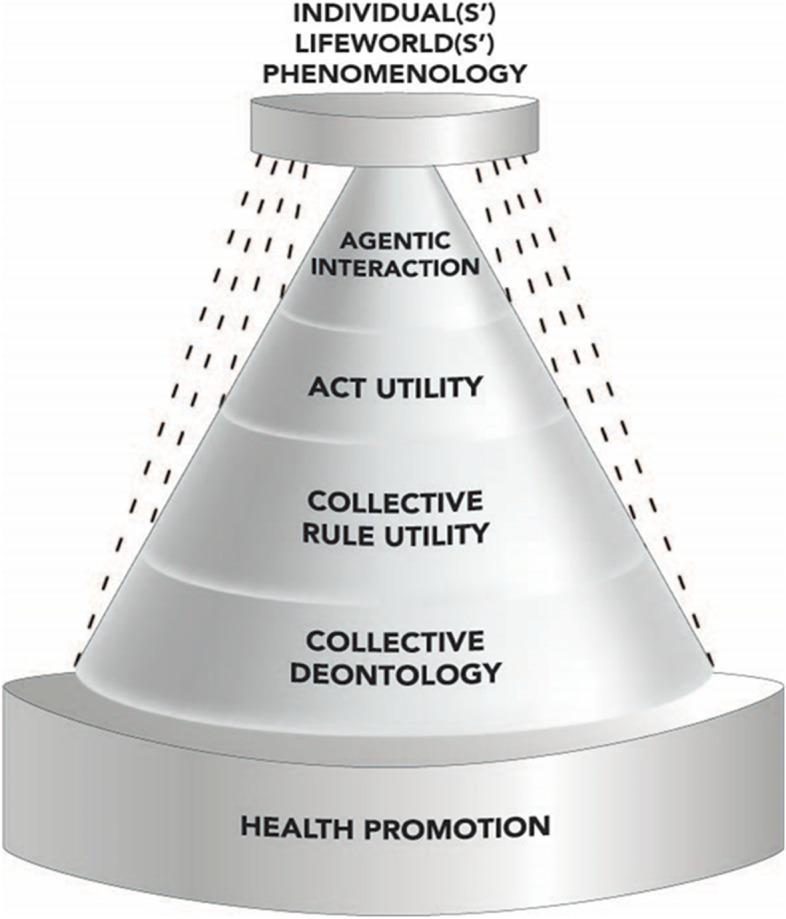
Diagrammatic representation of the inter-related and expanding aspects of individual agents’ lifeworlds, and phenomenological subjectivity contributory to the agentic interactions and act utility, and the rule utility and deontology of the collective as served by the disciplines and practices of health promotion (see text for detailed explanation)

We posit that this framework is applicable to and for health promotion. As shown in Fig. 1, important to recognize is that the “point of engagement” at which duties, rules and actions become directly focused is the individual and individuals-in-collective(s). Thus, duties, rules and acts should reflect and regard the values, needs of communities served, and (as shown in Fig. 2) these communities should be sensitive and responsive to the needs and values of those individuals that are constituent – and contributory to its collective health.

Simply put, a healthy community is not comprised of “unhealthy” individuals; and thus, the health needs and values of a collective of individuals are fundamental to promote in any genuine endeavor toward community health. Prima facie, this is both rational, and ethically justifiable in its relative Heraclitan balance of attempting to acknowledge and serve the entirety and its parts (see [36]). Yet, despite its ethical justifiability, this approach also generates questions, if not problems when determining which individuals, and what needs and values to accommodate and prioritize.

Simple commutative justice (viz.- the allocation to each that to which they are ‘entitled’) generally presumes abundant resources, and does not explicitly define the grounds or methods for defining and determining such entitlement. This is especially true when individual phenomenological experience (and the needs and values such experience both fosters and reflects). Therefore, writ both large (i.e.- on a collective scale) and small (i.e.- on an individual level), some form of distributive justice must be employed. Here too, relative balance of liberty and difference (viz.- *maximin* and/or *minimax*) principles should be obtained, although a purely Rawlsian [37] approach might be regarded as less than effective and therefore not efficient in defining those individuals and constructs to be prioritized; and Thagard type equilibrium [17], while effectively utilitarian – and perhaps compatible with phenomenological factors in its cognitive orientation – while efficient, may be seen as biasing.

We opine that a modified form of Pierek synthesis [38], which brings together – with maximized situational/circumstantial effectiveness – both “luck” and social” dimensions of egalitarian allocation may provide a viable, and valuable approach for ethical foundations to guide health promotions. Toward such goals we believe that a dialectic process toward developing a true “synthesis” of (1) egalitarian constructs, (2) individual and collective needs and values identification and prioritization, and (3) extant, revised, and de novo enterprises and resources to accommodate needs, and allocate goods, and services.

To be sure, such an undertaking will require considerable dedication of funding, personnel, and administrative/policy support; and description of viable tactical methods for achieving and sustaining this strategic model in-practice are beyond the scope of this chapter. However, despite the investment required, we believe that the collective and individual return(s) of such endeavor are important and worthwhile to establish and sustain health promotions systems and practices on the twenty-first century global stage. A subject-oriented—not simply individualized—health promotion, which appreciates subjectivity on a fundamental level will also contribute to the development of diversity and democratic processes. Our ongoing work remains dedicated to these efforts.

## Data Availability

Not applicable.

## References

[1] Elkeles T. Evidenzbasierung und Evaluation in der Gesundheitsförderung. In: Gesundheitsförderung stärken: kritische Aspekte und Lösungsansätze. Wien: facultas wuv universitätsverlag; 2008. p. 61–78.

[2] Klotter C. Warum wir es schaffen, nicht gesund zu bleiben. Eine Streitschrift zur Gesundheitsförderung. München: Reinhardt; 2009.

[3] Brunnett R. Foucaults Beitrag zur Analyse der neuen Kultur von Gesundheit. In: Anhorn R, Bettinger F und Stehr J (Eds). Foucaults Machtanalytik und soziale Arbeit. Eine kritische Einführung und Bestandsaufnahme. 1st ed. Wiesbaden: VS Verl. für Sozialwiss (Lehrbuch, 1); 2007. p. 169–184.

[4] Hensen P, Kölzer C (2011). Die gesunde Gesellschaft.

[5] Schröder-Bäck P. Ethische Prinzipien für die Public-Health-Praxis. Grundlagen und Anwendungen. Frankfurt, New York: Campus Verlag; 2014.

[6] Fulford KWM (2008). Values-based practice: A new partner to evidence-based practice and a first for psychiatry?. Mens Sana Monogr.

[7] Waters D, Sierpina V (2016). Goal-directed health care and the chronic pain patient: a new vision of the healing encounter. Pain Phys.

[8] Röhrich C, Krüger E, Kohls N. Gefühl und Vernunft als leibliches Erleben – Zur Relevanz von Subjektivität für anthropologische und ethische Bezüge im Kontext von Gesundheitsförderung. Internationale Zeitschrift für Philosophie und Psychosomatik. 2018;1.

[9] Droysen JG. Historik: Vorlesungen über Enzyklopädie und Methodologie der Geschichte. Stuttgart: Fromann-Holzboog, 1977 [1858]:22, 150f.

[10] Dilthey W. Einleitung in die Geisteswissenschaften“. Volume 2. B.G. Stuttgart: Teubner Verlagsgesellschaft; Göttingen: Stuttgart Vandenhoeck & Ruprecht; 1883.

[11] Giordano J, Abramson K, Boswell MV (2010). Pain assessment: Subjectivity, objectivity, and the use of neurotechnology. Pain Physician.

[12] Welt T, Grönemeyer HW, Kobusch T, Schott H, Grönemeyer D, Welt T (2008). Über den Begriff der Gesundheit in der daseinsanalytischen Medizin. Gesundheit im Spiegel der Disziplinen, Epochen, Kulturen.

[13] Engel G (1977). The need for a new medical model: a challenge for biomedicine. Science.

[14] Franke A. Modelle von Gesundheit und Krankheit. 3rd, revised ed. Bern: Huber; 2012.

[15] Giordano J, Becker K, Shook JR (2016). On the “neuroscience of ethics” – Approaching the neuroethical literature as a rational discourse on putative neural processes of moral cognition and behavior. Neurol Neuromed.

[16] Svenaeus F (2000). The Hermeneutics of Medicine and the Phenomenology of Health.

[17] Thagard P (2019). Mind—Society: From Brains to Social Sciences and Professions.

[18] Schmitz H (2014). Kurze Einführung in die Neue Phänomenologie.

[19] Gadamer H-G. Über die Verborgenheit der Gesundheit. Aufsätze und Vorträge. 1st ed. Berlin: Suhrkamp (MedizinHuman, 10); 2010.

[20] Horner JS (2000). For debate The virtuous public health physician. J Public Health Med.

[21] Rothstein MA (2004). Are traditional public healthstrategies consistent with contemporary American values?. Temple Law Rev.

[22] Holland S (2007). Public Health Ethics.

[23] Nixon S, Forman L. Exploring synergies between human rights and public health ethics: A whole greater than the sum of its parts. BMC Int Health Hum Rights. 2008;8(2). 10.1186/1472-698X-8-2 PMC2268657 18237409

[24] Roberts MJ, Reich MR (2002). Ethical analysis in public health. Lancet.

[25] Schmitz H (2016). Ausgrabungen zum wirklichen Leben.

[26] Böhme G. Ethik leiblicher Existenz. Über unseren moralischen Umgang mit der eigenen Natur. 1st ed. Frankfurt am Main: Suhrkamp Verlag (Suhrkamp Taschenbuch Wissenschaft, 1880); 2008.

[27] Kohls, NB. Außergewöhnliche Erfahrungen – blinder Fleck der Psychologie? Eine Auseinandersetzung mit außergewöhnlichen Erfahrungen und ihrem Zusammenhang mit geistiger Gesundheit. Available: Freiburg (Breisgau), Univ., Diss., 2004. Münster: Lit (Psychologie des Bewusstseins / Abt. A, Texte, Bd. 2); 2004.

[28] Julmi C, Scherm E (2012). Subjektivität als Ausdruck von Lebendigkeit. Zeitschrift für Philosophie und Psychosomatik.

[29] Schmitz H. Selbst sein. Über Identität, Subjektivität und Personalität. Originalausgabe. Freiburg, München: Verlag Karl Alber; 2015.

[30] Schmitz H, Müllan RO, Slaby J (2011). Emotions outside the box—the new phenomenology of feeling and corporeality. Phenomenol Cogn Sci.

[31] Schmitz H (2019). Wie der Mensch zur Welt kommt.

[32] Flanagan O, May L, Friedman M, Clarj A (1996). Ethics naturalized: ethics as human ecology. Mind and Morals: Essays on Cognitive Science and Ethics.

[33] Maricich Y, Giordano J (2007). Pain, suffering and the ethics of pain medicine: Is a deontic foundation sufficient?. Am J Pain Manag.

[34] Giordano J. Neuroethics: Coming of age and facing the future. In: Giordano J, Gordijn B, editors. Scientific and Philosophical Perspectives in Neuroethics. NY: Cambridge University Press; 2010. xxv-xxix.

[35] Applewhite M, Giordano J, Girton J, Procaccino J. Rapid Notes for Research and Clinical Military Medicine. Bethesda: Department of Defense Medical Ethics Center; 2022.

[36] Kahn CH (1981). The Art and Thought of Heraclitus.

[37] Rawls J (1971). A Theory of Justice.

[38] Pierek R, Robeyns I (2007). Resources versus capabilities: Social endowments in egalitarian theory. Polit Stud.

